# Book Review of “Workflow Optimization in Radiation Oncology: From Theory to Clinical Implementation” By Colleen J. Fox and Reshma Munbodh (Medical Physics Publishing)

**DOI:** 10.1002/acm2.70565

**Published:** 2026-04-09

**Authors:** Emily Y. Hirata

**Affiliations:** ^1^ Department of Radiation Oncology, Division of Medical Physics University of California, San Francisco San Francisco California USA

## 1 OVERVIEW

Today's clinical practice of radiation oncology relies heavily on the coordinated efforts of multiple teams in an environment of complex technologies and systems. As physicists, we devote significant effort and attention on testing and evaluating treatment planning and delivery systems, their interoperability, and the standardization of information exchange between systems. Errors, however, often arise from breakdowns in communication and suboptimal workflows equipped with inadequate controls or direction. Incorrect allocation of resources can further increase errors, raise costs, and exacerbate burnout.

The field of operations management focuses on overall planning, direction, and control of the processes that convert inputs (labor, equipment) into outputs (goods, services). With a history of application across various sectors, operations management is used in supply chain logistics and lean manufacturing in automobile production and, in recent years, has found application in radiation oncology. This book introduces the theory of operations management and its application to practical challenges prevalent in radiation oncology practices. Edited by Colleen J. Fox and Reshma Munbodh, *Workflow Optimization in Radiation Oncology: From Theory to Clinical Implementation* provides a much‐needed roadmap for navigating operational complexities, providing a valuable framework for physicists and other radiation oncology professionals involved in continuous improvement, project management, and resource allocation. The book emerged as a byproduct of the 2024 Summer School of the American Association of Physicists in Medicine (AAPM), where the AAPM dedicated an entire summer school program to this critical topic. The expert faculty authored the book and used its content as the foundation for teaching this well‐received summer school, reinforcing its value as an essential resource for the field.

## 2 CONTENTS AND HIGHLIGHTS

This book is 275 pages and contains a total of 16 chapters divided into 2 sections: Theory and Clinical Implementation. The first six chapters cover operations management theory and application (chapter 1), continuous improvement and project management (chapter 2), workflow robustness (chapter 3), standardization for workflow efficiency (chapter 4), measuring a system (chapter 5), and modeling and controlling a system (chapter 6). The remaining 10 chapters delve deeper into the practical application of operations management focused on specific radiation oncology situations. These include resource allocation in radiation oncology (chapter 8), optimizing time management (chapter 9), workflow considerations for implementing AI‐based auto‐contouring (chapter 10), quality assurance techniques for enhanced radiation oncology workflows (chapter 11), enhanced treatment planning implementation strategies (chapter 12), workflows for custom clinical software (chapter 13), developing and optimizing an adaptive radiotherapy program (chapter 14), effective communication skills for medical physicists (chapter 15), and change management (chapter 16).

## 3 CRITICAL ASSESMENT

This book effectively grounds readers in fundamental theory and principles before applying those methodologies to address common and modern challenges. First, chapter 1 begins with the basics of building process maps, introducing Little's Law which defines the relationship between a process’ throughput time, work‐in‐process, and throughput rate, and an overview of strategies for managing bottlenecks. For example, the book illustrates how a radiation oncology dosimetry team's preference to delay contouring tasks (waiting until they can start treatment planning instead of working in parallel with other teams) can significantly increase a patient's overall treatment time. This example illustrates how one team's preference for batching their tasks can impact the workflow for other connected processes, and ultimately the overall patient's total throughput time.

The book also explores continuous improvement, introducing guiding principles and explaining the standard DMAIC (Define, Measure, Analyze, Improve, Control) problem‐solving methodology. The author walks through a melanoma referral project where DMAIC methodology was employed and provided illustrative context for its application. One insightful tool was the PICK chart that pairs effort against benefit as a framework for evaluating different interventions: Possible (low effort, low benefit intervention), Implement (low effort, high benefit interventions), Challenge (high effort, high benefit interventions), and Kibosh (high effort, low benefit interventions). Because quality improvement initiatives are often project‐based, the book emphasizes the importance of planning, organization, and clear communication. Managing a project, particularly larger‐scale projects involving multiple teams, can be overwhelming and lead to poor execution if one is not equipped with tools or training in project management.

Beyond organizational processes, the book also dedicates attention to optimizing personal effectiveness. Chapter 9 delves into personal resource management, offering toolkits for structure (personal task management systems), protection (time blocking), and adaptability. It highlights the importance of time blocking to maintain focus and prevent burnout, ultimately contributing to both personal and team well‐being.

Each chapter is well written with accompanying charts and illustrations that are clear and well‐articulated. The content is presented in a cohesive and concise style that is easy to read and follow. The authors of each chapter have direct clinical experience and applied the presented methodologies in their own practice.

Radiation oncology is increasingly complex, with reirradiation, increasing use of SBRT and gating, and multi‐modality treatment strategies that require nuance care coordination within all disciplines. The hybrid environment can increase communication challenges and reduce visibility through conventional methods, highlighting the need for robust and clear processes. Designing workflows and developing processes is not a static practice, and it is increasingly critical for medical physicists to be able to assess and change processes that are no longer effective. While the field of radiation oncology is rapidly evolving, *Workflow Optimization in Radiation Oncology* provides a solid foundation in operations management principles that can be applied to many clinical scenarios, making it an essential tool for any radiation oncology team committed to continuous improvement and operational excellence.

